# Economic impact of childhood/adolescent ADHD in a European setting: the Netherlands as a reference case

**DOI:** 10.1007/s00787-013-0477-8

**Published:** 2013-10-29

**Authors:** Hoa H. Le, Paul Hodgkins, Maarten J. Postma, Jennifer Kahle, Vanja Sikirica, Juliana Setyawan, M. Haim Erder, Jalpa A. Doshi

**Affiliations:** 1 PharmacoEpidemiology & PharmacoEconomics, University of Groningen, Antonius Deusinglaan 1, 9713 Groningen, The Netherlands; 2 Global Health Economics and Outcomes Research, Shire, 725 Chesterbrook Boulevard, Wayne, PA 19087 USA; 3 BPS International, 3830 Valley Centre #705 PMB503, San Diego, CA 92130 USA; 4 General Internal Medicine, University of Pennsylvania, 1222 Blockley Hall, Philadelphia, PA 19104-6021 USA

**Keywords:** Attention-deficit/hyperactivity disorder, Children, Adolescents, Cost of illness, Societal costs

## Abstract

Attention-deficit/hyperactivity disorder (ADHD) is a highly prevalent psychiatric disorder in children/adolescents. This study reviews available European-based studies of ADHD-related costs and applies the findings to the Netherlands to estimate annual national costs for children/adolescents from a societal perspective. A systematic literature search was conducted for primary studies in Europe, published January 1, 1990 through April 23, 2013. Per-person cost estimates were converted to 2012 Euros and used to estimate annual national ADHD-related costs based on the Dutch 2011 census, ADHD prevalence rates, family composition, and employment rates. Seven studies met the inclusion criteria. The average total ADHD-related costs ranged from €9,860 to €14,483 per patient and annual national costs were between €1,041 and €1,529 million (M). The largest cost category was education (€648 M), representing 62 and 42 % of the low- and high-value overall national estimates, respectively. By comparison, ADHD patient healthcare costs ranged between €84 M (8 %) and €377 M (25 %), and social services costs were €4.3 M (0.3–0.4 %). While the majority of the costs were incurred by ADHD patients themselves, €161 M (11–15 %) was healthcare costs to family members that were attributable to having an ADHD child/adolescent. In addition, productivity losses of family members were €143–€339 M (14–22 %). Despite uncertainties because of the small number of studies identified and the wide range in the national cost estimates, our results suggest that ADHD imposes a significant economic burden on multiple public sectors in Europe. The limited number of European-based studies examining the economic burden of ADHD highlights the need for more research in this area.

**Electronic supplementary material:**

The online version of this article (doi:10.1007/s00787-013-0477-8) contains supplementary material, which is available to authorized users.

## Introduction

The burden of psychiatric disorders in developed countries is large, representing up to 40 % of all brain diseases (mental and neurological disorders) and 14 % of all diseases [38]. A recent analysis has reported that the total cost of psychiatric disorders in Europe was approximately €432 billion, of which direct healthcare costs, direct non-medical costs and indirect costs represent 36, 12, and 52 %, respectively, of the total [37]. This figure was higher than previously estimated [2], but may still be a conservative estimate given that not all costs were included because of a lack of data on many disorders or cost items [37]. Costs of attention-deficit/hyperactivity disorder (ADHD) in children and adolescents were included in the analysis [37], but the authors only assessed direct healthcare costs and direct non-medical costs. Moreover, total costs were estimated for Europe (represented by all members of the European Union plus Iceland, Norway, and Switzerland). Thus, the reported costs of ADHD did not capture the broad societal impact of the disease, particularly at the national level, which may be important in country-specific policy decisions.

ADHD is a chronic behavioural disorder with onset in childhood that often persists into adulthood [29, 46]. It is characterized by symptoms of inattention, hyperactivity, and impulsivity [1, 61]. Furthermore, the symptoms can cause significant distress and impairment, and are pervasive (i.e., impairment is observed in many areas of life, such as school performance, social function, and occupational achievement). The two main international diagnostic criteria used clinically are the International Classification of Diseases, 10th Edition (ICD-10) [61] and the Diagnostic and Statistical Manual of Mental Disorders (DSM), 5th Edition, Text Revision [1]. The former is predominately used by European clinicians, while the latter is widely used in the United States (US), as well as by some European physicians [21]. The prevalence rates of ADHD in children and adolescents are high with estimates of 5.3 % [45] to 5.9 % [59] worldwide and 4.6 % for Europe [45]. Similar prevalence rates of approximately 5 % were observed for adults with ADHD worldwide [59].

Impairments associated with ADHD are multi-faceted, with outcomes such as academic failure, self-esteem problems, and interpersonal relationship difficulties [11, 57, 60]. People with ADHD are also more prone to injuries and accidents, including serious injuries and traffic accidents, than those without ADHD [18, 31, 51]. Other associations with ADHD include substance abuse problems and interactions with the criminal justice system [32, 48, 52]. Not surprisingly, ADHD is associated with a reduction in overall and health-related quality of life [7, 25]. Moreover, the negative impact of ADHD may extend to family members, causing increased levels of stress, depression, marital discord, and reduced health-related quality of life for the family members of patients with ADHD [30, 33, 34]. While these long-term outcomes are worse for people with ADHD compared to those without, treatment using pharmacological, non-pharmacological, or combined approaches (e.g., medication and behavioural therapy) can ameliorate some of the negative impacts of the disorder [44, 50]. Treatment of ADHD may also have benefits beyond the individual. Comparing ADHD medication use and incidences of criminality among patients with ADHD, Lichtenstein et al. [28] have reported that the rates of criminality were lower during periods of medication use compared to periods off medication in the same patients.

Children and adolescents with ADHD consume more healthcare resources and consequently incur more healthcare costs compared to those without ADHD [15, 27, 47]. The financial impact of ADHD, however, affects many facets of a child’s life. A recent analysis of the economic burden of ADHD in the US highlighted that the majority of total disorder costs lie outside the healthcare sector, and because ADHD often persists into adulthood, costs of ADHD in the adult population are also substantial [10]. Furthermore, the financial burden of ADHD is not restricted to patients alone. Family members also incur significant costs, in the form of increased healthcare costs and productivity loss [4, 10, 54].

Similar cost-of-illness studies are scarce in Europe, and there is a lack of understanding of the economic burden of ADHD in Europe. The purpose of this study is to review the available evidence and to apply the findings to quantify the societal economic burden of ADHD in a European setting, using the Netherlands as a reference case. The focus of the present study is on the costs of ADHD in children and adolescents because there were no studies on adult ADHD-related costs identified.

## Methods

### Systematic literature search

A systematic review was conducted using guidelines from the Cochrane Handbook for Systematic Reviews of Interventions [17]. Five large databases (MEDLINE, EMBASE, ERIC, HEED, and PsycINFO) were searched for articles published from January 1, 1990 through April 23, 2013 using the following abstracted search strategy: (terms describing ADHD) AND [(terms describing cost analysis or economic impact) OR (terms describing areas of cost due to ADHD)] (See Supplementary Table 1 for search term details). Search dates were between June 1, 2011 and April 23, 2013.

**Fig. 1:**
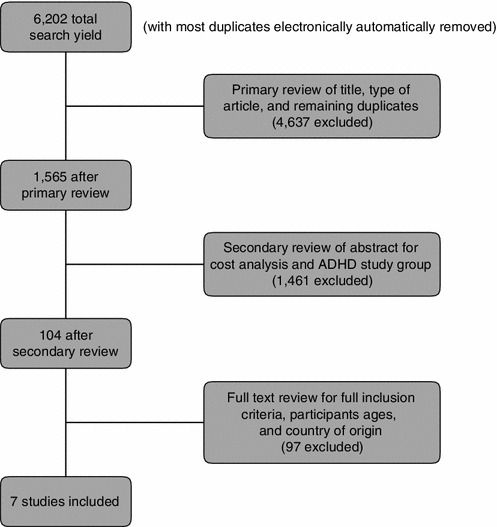
Flow chart describing the systematic literature review process. *ADHD* attention-deficit/hyperactivity disorder

The screening process is outlined in Fig. 1. Two researchers reviewed the citations and abstracts independently and agreed on the studies to be included. Any discrepancies were resolved upon review of the full text by the two reviewers and/or consultation with a third researcher. Articles retained for analysis (any language) included all those that were classified as original research studies of human participants conducted in Europe that monetized ADHD-related outcomes, either the excess costs of a population with ADHD compared with those of non-ADHD controls or costs specifically related to an ADHD diagnosis. All studies were original research and peer-reviewed; meta-analyses, case studies, editorials, opinion papers, and review articles were excluded. Studies in which costs related to ADHD were not analysed separately from other disorders were excluded. Study characteristics and cost measurements for included studies were extracted and tabulated by two researchers independently (all discrepancies were resolved between these two researchers), and fact-checked by a third researcher.

### Extraction and calculations of per-person ADHD-related costs

Some calculations and/or adjustments of the data reported in the studies were required to align the information presented in the papers to our framework. From the Wehmeier et al. [58] study, the total cost of treatment for hyperkinetic disorder in Germany for the age group <15 years was reported to be €287 million in 2006. Per-patient annual costs were computed by dividing the aggregate annual national costs by an estimated ADHD population size for this age group, which was derived using sex-specific prevalence rates [22, 36] and the applicable population size, matching as closely as possible the age distribution and census year with the year of study [22, 36]. From these calculations, the estimated ADHD population (aged <15 years) was 406,787, giving an estimated per-person cost of €706. After adjustments to Dutch 2012 euros, this figure becomes €856, which is comparable to the inflated estimate of €798 from Schöffski et al. (another German study on the healthcare cost of ADHD) [35, 49, 55, 58]. In the Myren et al. [35] study, direct and indirect costs at baseline were reported for the treatment and placebo groups 10 weeks prior to an ADHD treatment period. As these baseline values represented costs before initiation of ADHD treatment, the data from both groups were combined. For this study, the productivity loss component of the combined indirect costs was extracted from data gained through written communication with the corresponding author. All 10-week costs were then extrapolated to annual costs.

All cost estimates reported in foreign currencies were converted to Euros for the matching year using gross domestic product per capita purchasing power parity provided by the Organisation for Economic Co-operation and Development [40]. These estimates were then inflated to 2012 levels using the Harmonized Index of Consumer Prices (HICP) for Education, Health, and Social Protection for the Netherlands as provided by the Federal Reserve Economic Data from the Federal Reserve Bank of St. Louis [14].

### Estimation of annual national ADHD-related costs

Costing data were drawn from all European studies meeting our inclusion criteria. From the included studies, per-patient cost estimates were used to calculate national ADHD-related costs for the Netherlands, the European reference country for this study. This study did not assess or adjust for differential study quality or characteristics beyond those specified in the inclusion criteria (original, peer-reviewed research studies), including inherent statistical limitations of studies with a small sample size. As a result, point estimates for national costs were not calculated but instead the lowest and highest per-patient cost categories for each subcategory were identified (when more than one data source was available) and then used to estimate a total national ADHD-related cost range.

The number of children and adolescents aged 7–17 years with ADHD in the Netherlands was estimated by multiplying the 2011 Dutch census estimates for this age group with the estimated prevalence of ADHD in the Netherlands. The only published ADHD prevalence estimate (2.1 %) for the Netherlands, however, is limited to prevalence of pharmacologically treated children and adolescents with ADHD [20]. Use of this ‘treated ADHD’ prevalence rate would likely result in an underestimate of the national costs of ADHD in the Netherlands as undiagnosed and/or untreated children and adolescents with ADHD would also incur healthcare- and education-related costs. Hence, in our base-case analysis, we used a prevalence rate of 4.8 % reported for German children and adolescents aged 7–17 years [22]. Data from the German study were used as a proxy for the Netherlands because of the social, economic, and cultural similarities between the two countries. Moreover, the German prevalence rate was similar to the reported ADHD prevalence for Europe (4.6 %) [45]. In the sensitivity analysis (described later in this section), we used the treated ADHD prevalence rate (2.1 %) reported in the Netherlands.

The estimation of the number of family members incurring costs related to ADHD required additional multiplication factors to determine the population size incurring the costs. Because the costs of healthcare and productivity losses to family members of patients with ADHD were applicable to adult members and not to other siblings of similar age in our analysis, a multiplication factor representing the average number of adults in households with children in the Netherlands was needed. This adult member multiplication factor was estimated to be 2.26 and was derived from the reports ‘SF1.1 Family Size and Composition’ [41] and ‘SF1.4: Population by Age of Children and Young Adults, and Youth-Dependency Ratio’ [42] by the Organisation for Economic Co-Operations and Development (OECD). The percentages of couple families and single-parent families of all households in the Netherlands were combined with the average household sizes of couples with children and single-parents with children to derive the average size of Dutch households with children [41]. The proportion of adults in these households was calculated using the youth-dependency ratio [42]. This factor was used to calculate healthcare costs to adult members living in households with a child/adolescent with ADHD. The product of the employment rate in the Netherlands for 2011 (0.77) and the adult member multiplication factor (2.26) was used to determine the number of family members whose work productivity would be affected by a child/adolescent with ADHD [12, 41, 42].

For each cost category, the low and high per-patient cost estimates were used to establish an annual national cost range for that category. For some categories such as healthcare costs to family members or education and social services costs to patients with ADHD, however, only one estimate was available. In these cases, the same estimate was used to calculate the low and high values in the range of total national costs. While there were two studies that investigated costs of education for patients with ADHD, these studies reported different aspects of education costs [8, 55]. De Ridder and De Graeve [8] included only the costs to the parents from extra school lessons for their child/adolescent with ADHD, whereas Telford et al. [55] examined various elements of costs to the education system such as services and time commitment from teachers, social workers, counsellors, educational psychologists, etc. but did not include extra school costs to the parents [55]. Thus, the sum of the two estimates was used to establish one overall per-patient education cost.

### Sensitivity analysis

The methods described above are for the base-case analysis. In economic modelling, the base-case represents the most likely assumptions on model structure and/or input parameters/data. Sensitivity analyses are also recommended to evaluate the robustness of the results as well as to assess different relevant scenarios. Thus, in addition to the base-case analysis, three sensitivity analyses were performed to vary key parameters in our base-case analysis. In the first scenario, the ADHD prevalence rate (4.8 %) was changed to the diagnosed and treated prevalence rate (2.1 %) for the Netherlands [20].

The second scenario included a study by Petrou et al. [43], which did not meet the inclusion criteria for the base-case analysis because it reported an aggregated estimate for healthcare, education, and social services. The Petrou study is similar to the Telford study [55] in that both studies estimated costs of ADHD in the UK and the same cost categories were investigated, albeit an aggregated estimate was reported in the former whereas disaggregated estimates were provided in the latter. From these similarities, we assumed that the merged estimate from the Petrou study could be disaggregated into each cost category according to the distribution profile that was observed in the Telford study. As a result of the disaggregation, new per-person low estimates for healthcare, education, and social services costs to the patients with ADHD were used in the national cost calculation.

The last scenario included a study by Knapp et al. [26] that investigated the connections between attention deficit problems in childhood and future earnings in adulthood. The authors used the 1970 British Cohort Study to collect data on attention deficit problems at age 10 years (assessed by the Connors Teachers Rating Scale) and employment activities at age 30 years. A probit regression model was used to assess the influence of childhood variables on future employment and occupational status. Combining the result of the probit model with an analysis on earnings of those in employment, the authors calculated the impact of attention deficit problems in childhood on expected earnings in adulthood. The findings from this study suggested that attention deficit problems at age 10 years were associated with lower employment rates, worse jobs, and lower wages when employed, leading to overall lower expected earnings at age 30 years. The authors reported £36 and £61 in weekly income losses for males and females, respectively. Annual income losses were calculated using an estimated average of working weeks per year in the Netherlands (45.2 weeks) [39]. Based on the applicable population sizes of males and females, a weighted average for annual income losses was estimated to be €4,486 after currency conversion and inflation to 2012 level. In the sensitivity analysis, we included this future income loss as an additional cost category for children and adolescents with ADHD.

## Results

From an initial yield of 6,202 citations, a primary review of the titles, type of articles, and remaining duplicates excluded 4,637 studies (Fig. 1). Upon a review of each study abstract to identify the ADHD study population and cost analysis, another 1,461 studies were excluded, leaving 104 potential studies. After a full-text review, seven studies on cost of ADHD in children and adolescents met full inclusion criteria for base-case analysis [5, 8, 16, 35, 49, 55, 58] and two studies were retained for sensitivity analysis [26, 43]. Study characteristics of those used in the base-case analysis are summarized in Table 1. The included studies were from five northern/western countries from the European Union (EU): Belgium, Germany, the Netherlands, Sweden, and the United Kingdom. Most studies were retrospective analyses with the exception of one prospective analysis [16] and one placebo-controlled clinical trial [35]. ADHD was diagnosed according to either ICD-10 or DSM 4th edition. Text revision criteria in all studies but one where the Connors Teacher Rating Scale was used [8]. Four studies used the total cost approach [35, 49, 55, 58] while three studies used the incremental cost approach [5, 8, 16] to identify ADHD-related costs. Last, most studies did not provide adjustment for possible confounders.

**Table 1:** Summary characteristics of studies included in the base-case analysis

Study	Country	Study design	Sample size	Age (years) of patients range or mean (SD)	ADHD diagnosis	Type of cost-of-illness approach	Cost categories	Cost year reported	Adjustment for confounders
De Ridder and De Graeve [8]	Belgium	Retrospective analysis of non-random sample of Flemish members of ADHD society	909	Range 1–18 ADHD mean 11.1 Non-ADHD mean 10.2	IOWA-CRS scale^a^	Incremental	Healthcare and education (patient)	2002–2003	Unadjusted^b^
Hakkaart-van Roijen et al. [16]	Netherlands	Follow-up analysis of patients treated by paediatricians for ADHD	145	Range 6–18 ADHD mean 10.5 (2.7) Non-ADHD mean 7.8 (1.0)	DSM-IV	Incremental	Healthcare (patient) Healthcare (family member) Productivity loss (family member)	2004	Unadjusted
Schöffski et al. [49]	Germany	Retrospective analysis from national accounts	225,000	<15^c^	ICD-10 F90	Total cost	Healthcare (patient)	2002	Unadjusted
Wehmeier et al. [58]	Germany	Retrospective analysis of insurance claims	n.r.	Range 0–85+ <15^d^	ICD-10 or DSM-IV-TR	Total cost	Healthcare (patient)	2006	Unadjusted
Myren et al. [35]	Sweden	Placebo-controlled clinical trial	99	Range 6–15	DSM-IV	Total cost	Healthcare (patient) Productivity loss (family member)	2005	Adjusted
Telford et al. [55]	UK	Retrospective analysis of Cardiff ADHD study (CLASS)	143	Range 12–18 14.06 (1.69)	ICD-10 or DSM-IV	Total cost	Healthcare, education and social services (patient)	2010	Unadjusted
Braun et al. [5]	Germany	Retrospective analysis of insurance claims	145,608	Range 6–17	ICD-10 F90	Incremental	Healthcare (patient)	2008	Unadjusted

*ADHD* attention-deficit/hyperactivity disorder; *DSM-IV-TR* Diagnostic and Statistical Manual of Mental Disorders, 4th Edition, Text Revision; *ICD-10* International Classification of Diseases, 10th Edition; *SD* standard deviation

^a^IOWA-Connors Rating Scale is a rating scale, not an ADHD diagnostic test. The cut-off score for determination of ADHD that was used in the study was 15

^b^Authors reported both adjusted and unadjusted cost estimates. Adjusted estimates were not used in the current analysis because these were merged estimates

^c^This age group contributed to 93 and 83 % of the total cost for boys and girls, respectively

^d^This age group represented 66 % of the study population. Cost analysis in the current study was restricted to this age group

Five cost categories, for which costing data were available, were identified. Three categories were costs attributable to the child/adolescent with ADHD themselves (healthcare, education, and social services) and two were costs to family members that were due to ADHD-related activities of a child/adolescent with ADHD (healthcare costs and productivity loss). A list of the cost components that were described in each of the studies is provided in Supplementary Table 2. A summary of per-patient and per-family member cost estimates for each study is presented in Table 2. All studies reported ADHD-related healthcare costs attributable to patients. The estimates were between €798 and €3,571. One study also measured healthcare costs to family members (€675) [16]. Two studies reported costs for education [8, 55] and produced a derived estimate of €6,085. One study also considered social services costs utilized by the patient (€40) [55]. Productivity loss to family members of patients with ADHD was estimated in two studies to be between €781 [35] and €1,845 [16] per family member.

**Table 2:** Annual per-person ADHD-related costs by categories, adjusted to 2012 Dutch Euros

Study	Annual per-person costs (2012 Dutch Euros)^a^				
	Child/adolescent ADHD patient			Family member	
	Healthcare	Education	Social services	Healthcare	Productivity loss
De Ridder and De Graeve [8]	€1,436	€56			
Hakkaart-van Roijen et al. [16]	€2,191			€675	€1,845
Schöffski et al. [49]	€798				
Wehmeier et al. [58]	€856				
Myren et al. [35]	€2,009				€781
Telford et al. [55]	€1,918	€6,085	€40		
Braun et al. [5]	€3,571				

*ADHD* attention-deficit/hyperactivity disorder

^a^These estimates were used to establish the low and high range for national cost estimates

### Base-case analysis

The low and high estimates from each cost category were used to derive a range for total annual national ADHD-related costs for the Netherlands (Table 3). Annual national ADHD-related healthcare costs for children/adolescents with ADHD ranged between €84 and €377 million, and ADHD-related healthcare costs by family members were €161 million (Table 3). ADHD-related education and social services costs were €648 and €4.3 million, respectively. Productivity loss by family members due to ADHD-related activities of the child/adolescent was estimated to range from €143 to €339 million. Summing the estimates across the categories gave a range for the total national annual ADHD-related costs of €1,041–€1,529 million.

**Table 3:** National ADHD-related costs for the Netherlands, adjusted to 2012 Dutch Euros

Cost category	Number of studies	Population of interest^a,b^	ADHD prevalence for age range^c^ (%)	Other multipliers	Population incurring cost	Per-person ADHD-related cost (2012 Dutch Euros)	National ADHD-related cost (2012 Dutch Euros; in millions)
Healthcare costs							
ADHD patients	7	2,199,000	4.80	–	105,552	€798–€3571	€84–€377
Family members	1	2,199,000	4.80	2.26^d^	238,325	€675	€161
Subtotal per patient^e^						€2,322–€5,095	€245–€538
Education costs							
ADHD patients							
Extra lessons	1	2,199,000	4.80	–	105,552	€56	€5.9
Education system	1	2,199,000	4.80	–	105,552	€6,085	€642
Subtotal per patient^e^						€6,141	€648
Social services costs							
ADHD patients	1	2,199,000	4.80	–	105,552	€40	€4.3
Subtotal per patient^e^						€40	€4.3
Productivity and income losses							
Productivity losses (family)	2	2,199,000	4.80	2.26^d^, 77 %^f^	183,511	€781–€1,845	€143–€339
Subtotal per patient^e^						€1,358–€3,208	€143–€339
Total per patient						€9,860–€14,483	€1,041–€1,529

*ADHD* attention-deficit/hyperactivity disorder

^a^The Dutch Census, 2011; Centraal Bureau voor de Statistiek

^b^Age range of population was 7–17 years

^b^Huss et al. [22]

^d^Average number of adults in Dutch households with children, rounded from 2.25789631487208

^e^Per-patient cost is not the sum of per patient (per patient and per family member) costs. It was calculated using the national costs and ADHD patient population

^f^Employment rate for the Netherlands (20–64 years); European Commission Eurostat Labour Force Survey, 2011

Total annual per-patient costs were calculated from the total annual national costs because the applicable population is not the same across the different cost categories. National costs to family members were divided by the ADHD patient population to estimate the costs to family members per ADHD patient instead of costs per family member as reported in the included studies. For example, annual ADHD-related costs of healthcare for the patients and the family members ranged from €2,322 to €5,095 per ADHD patient (Table 3). Productivity loss by family members ranged between €1,358 and €3,208 per patient. Annual costs for education and social services per patient were €6,141 and €40, respectively. This corresponded to an average total annual cost per child/adolescent with ADHD between €9,860 and €14,483.

**Fig. 2:**
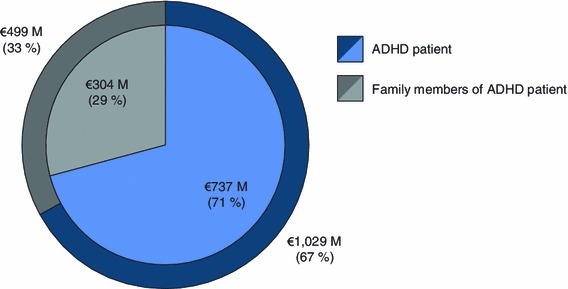
National ADHD-related costs (in millions) of ADHD patients and family members. The *inner circle* represents the low range estimate (€1,041 M) and the *outer circle* represents the high range estimate (€1,529 M). *ADHD* attention-deficit/hyperactivity disorder

**Fig. 3:**
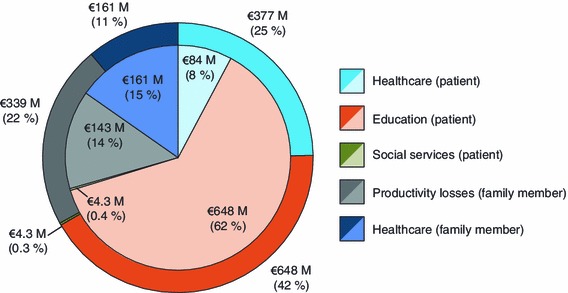
National attention-deficit/hyperactivity disorder-related costs (in millions) by cost categories. The *inner circle* represents the low range estimate (€1,041 M) and the *outer circle* represents the high range estimate (€1,529 M)

While the majority of the total cost was attributable to ADHD patients, costs to the family members accounted for 29–33 % of the total (Fig. 2). When considering the contributions of the different categories to the total cost, healthcare costs for ADHD patients were 8–25 % (Fig. 3). The most costly category was education, accounting for 42–62 % of the total. For family members, ADHD-related healthcare costs (11–15 %) and ADHD-related productivity losses (14–22 %) were comparable to healthcare costs for ADHD patients. Costs of social services for children/adolescents with ADHD constituted the smallest proportion (0.3–0.4 %).

### Sensitivity analysis

When using the diagnosed and treated ADHD prevalence rate for the Netherlands, the annual national cost range was reduced to between €455 and €669 million (Table 4). The inclusion of the disaggregated estimates from the Petrou et al. [43] study produced new low estimates for healthcare costs for the patient and costs to the education system and social services. This resulted in a new low estimate for the annual national cost of €372 million. A study that projected occupational consequences of children with ADHD when they become adults suggested that those diagnosed with ADHD in their childhood lost on average €4,486 per year in future earnings due to lower wages [26]. When including this study in a sensitivity analysis, the annual national costs of ADHD increased to between €1,514 and €2,002 million.

**Table 4:** Sensitivity analyses on national ADHD-related costs for the Netherlands

Analyses	National ADHD-related cost (2012 Dutch Euros; in millions)
Base-case	€1,041–€1,529
Sensitivity analyses	
Diagnosed and treated Dutch prevalence [20]	€455–€669
Inclusion of study with merged cost [55]	€372–€1,529
Inclusion of income loss as adults [26]	€1,514–€2,002

*ADHD* attention-deficit/hyperactivity disorder

## Discussion

To the authors’ knowledge, this is the first systematic review and analysis of ADHD cost of illness studies in Europe. Because of the small number of studies on cost of ADHD across Europe, the findings from the systematic review were pooled and then used to estimate the total annual national ADHD-related costs for a reference country, the Netherlands.

The results indicate that societal ADHD-related costs are approximately €1 billion annually for a country with a population of around 16 million. A similar study conducted in the US by Doshi et al. [10] reported that the total annual national cost of ADHD was estimated to be between $143 and $266 billion. There are several factors contributing to the difference in the national estimates between the two studies. First, the population size of the US is approximately 19-times larger than the Netherlands. Second, the reported prevalence rate of ADHD in children and adolescents in the US is higher than the prevalence rate used in this analysis [22, 56]. Last, there were more cost-of-illness studies of ADHD in the US compared with Europe. Thus, Doshi et al. were able to identify more cost categories such as costs to the justice system and more importantly costs of ADHD in adult patients. Costs to adult patients were major contributors to overall costs, representing approximately 73 % of the $143 and $266 billion estimates. These cost categories were not available in the present analysis. Hence, our estimates on only children/adolescents are limited in measuring the true societal impact of ADHD to the nation.

An important finding of our analysis was that the healthcare costs represented a small proportion (8–18 %) of the overall national ADHD-related costs in the Netherlands. The observation that the majority of the ADHD-related costs was outside of the healthcare sector has important policy-making implications given the social insurance systems in Europe [10]. In European social insurance systems, policy makers may find it beneficial to invest in improving the diagnosis and management of ADHD because this may offset costs in other sectors like education and the justice system, as well as productivity loss in the workforce. The economic impact of early diagnosis and treatment of ADHD in Europe, however, has not yet been investigated. This clearly warrants further research. In addition, a more complete understanding of the costs associated with ADHD in different public sectors may aid in budget decisions across the different sectors.

While healthcare is not the major cost driver of ADHD, it is the most investigated cost category. With the exception of patient education and productivity loss to family members, which were each included in two studies, the other cost categories were investigated by just a single study. The scarce evidence on ADHD-related costs other than healthcare highlights the need for more cost research in education, social services, and the financial impact of productivity loss by family members of children and adolescents with ADHD. This literature review also identified costs to the justice system, substance abuse treatment, and traffic accidents as areas that have been suggested to be negatively impacted by ADHD but for which no cost-of-illness studies are presently available in Europe. Furthermore, there is a complete lack of studies investigating cost of ADHD in adult patients in Europe, even though this cost category may represent the majority of the total cost, as in the US [10].

### Limitations and biases

The major limitation of the study was the small number of studies that were included in the analysis. Moreover, included studies did not report the same cost categories. As a result, most cost categories, other than healthcare costs, were reported by only one or two studies. This further limited the available evidence on overall costs of ADHD, which increased the uncertainties around the annual national cost estimates. Given the limited data, a point estimate for annual national cost was not provided, because high variance between studies was expected. Instead, low and high values for each cost category, when possible, were used to estimate a range for the annual national cost. Compounding of error was a concern also, especially considering the small sample of studies and extrapolation to large monetary values.

In addition to the small number of studies, there were other potential sources of bias. For instance, the seven included studies were from five different countries of the EU. Only one study was based in the Netherlands; of the remaining studies, three came from Germany and one each from Belgium, Sweden, and the UK. While all of these studies meeting the inclusion criteria were coincidentally based in northern/western countries of Europe, which share similar social-economic characteristics and cultural views on the treatment of ADHD, applying between-country cost estimates to the Netherlands may introduce bias given the differences in the healthcare, education, and social support systems across these countries. Whether this issue results in under- or over-estimation of the true overall national costs in the Netherlands is not clear.

Some insights may be gained by considering the contribution of the cost estimates from the single study conducted in the Netherlands to the overall national cost calculations [16]. The study by Hakkaart-van Roijen et al. [16] reported estimates for healthcare costs of patient, healthcare costs of family members, and productivity costs of family members. These estimates were represented in three of the five cost categories used to derive the high-range national costs, but was only used in one (healthcare costs of family members) of the five categories for the low-range national costs. Considering the contributions of the Dutch cost estimates to the overall national costs would suggest that potential between-country bias is lower for the high-range estimation and higher for the low-range estimation. Using the same line of argument, the true cost of ADHD in the Netherlands would more likely be closer to the estimated high-range national costs.

Other sources of heterogeneity may have arisen from differences in study designs, ranging from retrospective analyses to a clinical trial setting (Table 1). Furthermore, there were differences in ADHD diagnostic criteria used, type of methodological approach, and whether or not the estimates were adjusted for confounders. All these factors contributed to the overall uncertainty of the cost estimates.

### Potential factors for underestimation and overestimation

The prevalence rate of ADHD in children and adolescents in the Netherlands is not known. Consequently, a prevalence rate from Germany was used as a proxy, which has face validity but the consequence of which is that the true prevalence rate of ADHD in the Netherlands may be under- or overestimated. Nevertheless, even if a prevalence rate of 2.1 % for diagnosed and treated Dutch ADHD patients was used [20], as was done in the sensitivity analysis, the annual national costs of ADHD were still approximately €500 million. This estimate would likely be an underestimation as the true prevalence rate would be higher than the diagnosed and treated ADHD rate.

There are a number of other factors that suggest an underestimation of ADHD-related costs. For example, not all relevant cost categories were included, because of lack of data. There were no studies reporting costs to the justice system, substance abuse treatment, or traffic accidents. It is reasonable to assume that ADHD would impose costs in these areas [3, 32, 48, 52]. Underestimation may also occur because not all cost components were included in each of the cost categories.

Furthermore, the present study included only costs related to ADHD in children and adolescents. When considering potential future income losses to children with ADHD as adults in the work force, the annual national costs of ADHD in children and adolescents increased to approximately €1.5 billion. Costs of ADHD directly attributable to adult patients, however, were not evaluated because there were no European-based studies on the topic. Nevertheless, it is known that ADHD persists into adulthood [24] and the prevalence rate of ADHD in adults is comparable with the estimates for ADHD in children [9, 13, 59]. Thus, it is reasonable to assume that including costs to adult patients would increase the total cost of ADHD [6, 10, 19]. Moreover, the increase may be quite large considering the findings by Doshi et al. [10] that cost of ADHD in adults represented approximately 73 % of the total national cost.

There were also factors that may have contributed to an overestimation of the national cost estimates in the present analysis. Most studies that were used in the analysis reported cost estimates that were not adjusted for possible confounders. For example, it is well established that ADHD is commonly associated with comorbidities such as depression, anxiety, bipolar mood disorder, and conduct disorders [23, 53, 62]. Thus, cost estimates that do not adjust for comorbidities may overestimate the ADHD-specific costs.

In summary, the findings in this study offer a broad estimation of the societal costs of ADHD. While acknowledging the limitation of the analysis, the societal economic impact of ADHD for the Netherlands remains large. In addition, as the impairments associated with the disorder are multi-faceted and occur in multiple settings, societal costs associated with ADHD also impact multiple public sectors in addition to healthcare, with the majority of the costs lying outside of the healthcare sector.

## Electronic supplementary material

Below is the link to the electronic supplementary material.

Supplementary material 1 (DOCX 58 kb)

Supplementary material 2 (DOCX 67 kb)
