# Supplementary material for: Economic impact of childhood/adolescent ADHD in a European setting: the Netherlands as a reference case
Source: Eur Child Adolesc Psychiatry. 2013 Oct 29;23(7):587–98. doi: 10.1007/s00787-013-0477-8 (PMC4077218; doi:10.1007/s00787-013-0477-8)
Supplement: Supplementary file 1 — Supplementary material 1 (DOCX 58 kb) [file 787_2013_477_MOESM1_ESM.docx]

Supplementary Table 1. List of the search terms used.

| Outcomes of interest | Search terms to capture outcomes |
| --- | --- |
| ADHD subjects in all countries | ADHD or ADD or attention deficit or hyperkine* or TDAH or DAH or DAA |
| Cost analysis or economic impact | cost* or burden or econom* or expen* or budget or financ* or pharmacoeconom* |
| Productivity losses | productiv* or absen* or presen* or inefficien* or efficien* or work performance or job performance or work loss or lost work or human capital or income or employ* or unemploy* or socioeconomic status or SES or occupational scale or public assistance or disability benefit* or *term disability or workm?ns comp* or workers comp* |
| General services use | resource use or resource utili* or service* |
| Healthcare utilization | care or physician visit* or doctor visit* or physician encounter* or doctor encounter* or outpatient visit* or inpatient visit* or inpatient admission*or emergency or hospital* or day case or *care |
| Accidents | accident* or injur* or casualty or traffic behave* or traffic violation |
| Education | special education or special need* or Section 504 or IDEA or education plan or school psych* or remedial education or special class* |
| Drug abuse | drug rehab* or Substance-Related Disorders/ epidemiology/psychology/rehabilitation or treatment seek* or seeking treatment or substance abuse treatment facilit* or substance abuse program or (illicit drug or substance abuse or substance-related disorders and treatment) |
| Criminal behaviour | justice system or juvenile or incarcerat* or delinquen* or institution* or prison* or offender pathway or criminal behavior |

ADD, attention deficit disorder; ADHD, attention deficit/hyperactivity disorder; DAA, déficit de l'attention/activité in French or déficit de atención y actividad in Spanish; DAH, Déficit de l'attention/hyperactivité in French or déficit de atención con hiperactividad in Spanish; IDEA, Individuals with Disabilities with Education Act; TDAH, Trouble déficit de l'attention/hyperactivité in French or trastorno por déficit de atención con hiperactividad in Spanish.
