# Supplementary material for: Economic impact of childhood/adolescent ADHD in a European setting: the Netherlands as a reference case
Source: Eur Child Adolesc Psychiatry. 2013 Oct 29;23(7):587–98. doi: 10.1007/s00787-013-0477-8 (PMC4077218; doi:10.1007/s00787-013-0477-8)
Supplement: Supplementary file 2 — Supplementary material 2 (DOCX 67 kb) [file 787_2013_477_MOESM2_ESM.docx]

Supplementary Table 2. List of cost components identified by study.

| Study | Cost components listed in each study |
| --- | --- |
| De Ridder, 2006 | - Healthcare costs include visits to general practitioner and specialist, medication costs, hospitalizations, emergency room visits and paramedical costs such as physiotherapy, speech and language therapy, ergo therapy, psychotherapy and other therapies |
|  | - Education costs relate to extra school lessons |
| Hakkaart-van Roijen, 2007 | - Healthcare costs for the child/adolescent patients comprise services provided by general practitioners, ambulatory mental healthcare, psychiatric practices, out-patient psychiatrists, school doctors, paediatricians, medical specialists, physiotherapists, speech therapists, social workers and alternative health practitioners, as well as hospitalizations and medication |
|  | - Healthcare costs to family members include services provided by general practitioners, ambulatory mental healthcare, psychiatric practices, out-patient psychiatrists, company doctors, social workers, consultants for alcohol and drugs use, alternative health practitioners and medication |
|  | - Indirect costs to family members stem from productivity loss in the form of absence from work and reduced efficiency at work |
| Schöffski, 2008 | - Healthcare costs arise from hospitalizations, medication costs, outpatient care and rehabilitation such as physiotherapy, voice therapy, ergo therapy and music therapy |
| Wehmeier, 2009 | - Healthcare costs comprise inpatient treatments (e.g. hospital care and services of rehabilitation units), outpatient treatment (e.g. by primary care physicians, specialists and nursing care), medication costs and other treatment costs (e.g. emergency services, auxiliary medical services, occupational therapy, prevention and education, home care, treatment provided in foreign countries and administrative costs) |
| Myren, 2010 | - Healthcare costs comprise outpatient care as visits to child psychiatrists, psychologists, general practitioners, district nurses, etc. and inpatient care from hospitalizations that were due to accidents |
|  | - Indirect costs include loss of work days of the parent, time spent by school personnel in discussion with the parents, special aid resources in school and damages in the home from the child's behaviour |
| Telford, 2012 | - Healthcare costs comprise outpatient services (from general practitioners, community psychiatric nurses, psychologists, family therapists, child and adolescent mental health workers, art/drama/music therapy, health visitor/practice nurses, opticians), hospital-based care (from psychiatrists, paediatricians and speech therapists) and medication costs |
|  | - Education costs stem from special school needs, time commitment from teachers, social workers, counsellors, etc. and services of educational psychologists and school/doctors |
|  | - Social services relate to contact with social workers |
| Braun, 2012 | - Healthcare costs comprise outpatient services (services supplied to non-hospitalized patients by medical doctors or accredited psychotherapists in private practice), inpatient care (services supplied to hospitalized patients), pharmaceuticals (only prescriptions filled by patients), therapeutic devices and remedies (as prescribed by medical doctors but provided by other therapists, e.g. occupational therapists) and rehabilitation (often prescribed subsequent to a hospital stay to allow full recovery) |
